# Scientific Validation of "Clinically Proven" Claims: Compliance Focused and Global Regulatory Insights

**DOI:** 10.7759/cureus.100399

**Published:** 2025-12-30

**Authors:** Maheshvari N Patel, Nayan Patel

**Affiliations:** 1 Clinical Research, NovoBliss Research Private Limited, Ahmedabad, IND; 2 Pharmacology, Swaminarayan University, Ahmedabad, IND; 3 Clinical Research Operations, NovoBliss Research Private Limited, Ahmedabad, IND

**Keywords:** claim substantiation, clinically proven claims, nutraceuticals, regulatory guidelines, scientific credibility

## Abstract

The designation “clinically proven” represents a product's validation through scientifically rigorous, ethically sound, and regulatory-compliant clinical research. Establishing such a claim demands a systematic continuum from preclinical evaluations to well-designed randomized controlled trials (RCTs), multicentric validations, and real-world evidence studies that collectively demonstrate safety, efficacy, and reproducibility. This communication delineates the essential methodological and regulatory framework required to substantiate clinically proven claims. It underscores the critical role of robust RCTs, investigator-initiated trials, post-marketing surveillance, and global real-world studies encompassing diverse populations, standardized endpoints, and strict adherence to Good Clinical Practice (GCP) guidelines. Furthermore, it highlights the influence of geographical variability, investigator expertise, and environmental factors on study outcomes. The regulatory perspectives of key authorities, including the Central Drugs Standard Control Organization (CDSCO), the United States Food and Drug Administration (USFDA), the Therapeutic Goods Administration (TGA), and the European Medicines Agency (EMA), are discussed in relation to maintaining the authenticity and credibility of such claims. Emerging technologies such as artificial intelligence-assisted imaging, advanced instrumental analyses, and digital data monitoring are identified as pivotal tools enhancing evidence reliability. Ultimately, the concept of “clinically proven” extends beyond empirical validation, embodying scientific integrity, ethical transparency, and consumer trust at the intersection of innovation and regulatory compliance.

## Editorial

In the current era of evidence-based healthcare and cosmetic science, the term “clinically proven” has emerged as a cornerstone of scientific credibility and consumer confidence. It signifies that a product or formulation has been subjected to rigorous clinical evaluation under controlled and standardized conditions, yielding statistically and clinically meaningful, reproducible results. Yet, earning this designation extends far beyond marketing appeal; it reflects a comprehensive scientific endeavor grounded in ethical integrity, methodological precision, and regulatory compliance.

This editorial presents an overview of the scientific, methodological, and regulatory foundations essential for substantiating “clinically proven” claims. It highlights the critical role of sound study design, investigator expertise, geographical and population diversity, and adherence to established regulatory frameworks. Through these interconnected elements, the process of clinical validation transforms into a structured pathway that ensures not only product efficacy and safety but also reinforces public trust in scientific transparency and accountability.

Importance of "clinically proven" claims

A clinically proven claim substantiates that a product’s efficacy and safety are supported by robust empirical evidence generated through human clinical studies. Such validation holds multifaceted importance across scientific, regulatory, and commercial domains:

Clinically proven claims are essential because they strengthen the scientific credibility of a product by clearly distinguishing evidence-based formulations from those supported only by theoretical assumptions or anecdotal observations. They also play a critical role in regulatory compliance, as global health authorities require substantiated claims to protect consumers and prevent misleading or exaggerated product promotion. From a consumer perspective, rising health awareness has increased the demand for products backed by transparent scientific validation and clinically verified outcomes. In addition, clinically supported claims enhance market competitiveness, enabling products to gain an advantage in regulatory approvals, physician recommendations, and international market expansion. 

For example, a nutraceutical claiming “clinically proven to enhance iron absorption” should be supported by robust, multicentric human clinical trials demonstrating measurable improvements in bioavailability and relevant hematological parameters, with data generated from participants encompassing broad ethnic and geographical diversity.

Collectively, such claims embody the intersection of science, ethics, and trust, reinforcing the integrity of research-driven innovation and consumer assurance.

Scientific and clinical study designs required

The pathway to achieving a clinically proven claim involves a progressive series of scientifically structured studies, each addressing a unique aspect of safety, efficacy, and reproducibility.

In Vitro and Ex Vivo Studies

These are preliminary assessments conducted on cell lines, tissues, or biological models to identify mechanisms of action, absorption kinetics, and potential toxicities. For example, cytotoxicity assays and antioxidant potential tests serve as foundational evidence before human trials.

Pilot or Exploratory Studies

Small-scale, non-randomized, open-label studies are often conducted to test feasibility, dose tolerance, and measurement reliability. These studies help refine endpoints and guide the design of larger confirmatory trials.

Randomized Controlled Trials (RCTs)

The gold standard for clinical validation, RCTs involve random assignment of participants into treatment and control (or placebo) groups, ensuring unbiased outcomes. Double-blind designs further eliminate investigator and subject bias. For example, iron supplementation studies have demonstrated that oral iron formulations can significantly improve hemoglobin and serum ferritin levels over intervention periods of 8-12 weeks in randomized controlled trials, supporting efficacy-based claims when appropriately substantiated [1].

Longitudinal and Multicentric Studies

For products expected to have prolonged or regional efficacy, multicentric and longitudinal studies confirm reproducibility across demographics and time. Such studies enhance the generalizability of results and support claims in broader populations.

Geographical factors influencing clinical outcomes

Clinical responses to drugs, nutraceuticals, and topical formulations can vary significantly across geographical regions due to differences in genetic makeup, environmental conditions, diet, microbiome composition, and lifestyle habits.

Climatic Variations

Products targeting skin hydration or barrier function show varying efficacy in humid versus arid climates [2].

Ethnic and Genetic Variability

Pharmacogenomic differences influence the metabolism and bioavailability of both allopathic and plant-derived compounds.

Dietary Influences

Nutritional background can alter baseline biomarkers, affecting response to supplements [3].

Environmental Wxposure

UV index, pollution, and humidity impact the cutaneous response to topical formulations.

Therefore, regulatory authorities often require region-specific data or bridging studies when extrapolating data from one population to another. For instance, data generated in Europe may not directly apply to the Indian population due to differences in Fitzpatrick skin types and climatic conditions.

Series of studies required to substantiate a clinically proven claim

A clinically proven status typically demands a hierarchical validation pathway, integrating several study types that progressively strengthen the evidence base (Table 1).

**Table 1:** Required Series of Studies

Stage	Study Type	Purpose
Stage 1	Preclinical (In vitro/Ex vivo)	To understand mechanism, dose, and toxicity
Stage 2	Pilot Exploratory Study	To assess feasibility, endpoints, and tolerance
Stage 3	Randomized Controlled Trial	To establish safety and efficacy
Stage 4	Multicentric or Longitudinal Study	To confirm reproducibility across populations
Stage 5	Post-Marketing Surveillance	To ensure continued safety in real-world use

Each phase contributes a piece of the scientific validation puzzle. Cumulatively, these datasets justify the inclusion of the term “clinically proven” in product claims and marketing communication.

Regulatory framework and involved authorities

Various regulatory bodies and ethical frameworks play critical roles in validating and approving clinically proven claims. These ensure that data collection, subject safety, and claim substantiation adhere to globally accepted norms.

Indian Regulations

Indian Council of Medical Research (ICMR) Guideline and Responsible Conduct of Research: The ICMR mandates that all biomedical and health-related research in India must adhere to ethical guidelines, ensuring scientific integrity, participant safety, and responsible conduct throughout the research process. Researchers are required to obtain all relevant institutional and regulatory approvals before initiating a study, which may include clearances from the ethics committee (EC), scientific advisory committee (SAC), institutional animal ethics committee (IAEC), institutional biosafety committee (IBSC), institutional committee for stem cell research (IC-SCR), Genetic Engineering Appraisal Committee (GEAC), Review Committee on Genetic Manipulation (RCGM), Health Ministry's Screening Committee (HMSC), Central Drugs Standard Control Organization (CDSCO), or other applicable bodies depending on the nature of the work [4].

Investigators must also disclose any conflicts of interest and ensure transparency at every stage of the research. Furthermore, registration with the Clinical Trial Registry of India (CTRI) is mandatory for clinical trials and strongly encouraged for other study types to promote public accountability. By following these comprehensive ICMR guidelines, researchers uphold the highest standards of ethical practice, ensuring that the data generated is trustworthy, credible, and aligned with national expectations for responsible research conduct [4]. 

The Food Safety and Standards Authority of India (FSSAI), Under the Ministry of Health and Family Welfare: For any food, nutraceutical, or health supplement to carry ingredient-based, nutrient, nutritional, enhanced-function, or disease-risk-reduction claims, regulatory authorities require a thorough and credible scientific foundation. Such claims must be supported by evidence that links the specific ingredient or nutrient to the stated benefit, drawing from published scientific literature, official traditional texts, post-market data, consumer studies, cohort or retrospective analyses, and national or international epidemiological datasets. Additionally, validity studies demonstrating consensual, congruent, and concurrent support are essential. Claims relating to health promotion or disease-risk reduction must be backed by human efficacy and safety data, including not only controlled clinical trials but also nutra-epidemiological evidence. While structure-function claims may be permitted, they must be clearly worded, scientifically qualified, and easily understood by consumers, without implying treatment or cure. Claims suggesting drug-like effects, such as “prevents bone fragility in post-menopausal women”, or claims implied through product names, graphics, electrocardiogram (ECG) traces, or diagnostic symbols are strictly prohibited. For structure-function claims, population-specific guidance may be required, tailored to age groups, gender, or vulnerable populations. These principles ensure that nutritional and functional claims remain accurate, responsible, and grounded in robust scientific evidence, ultimately protecting consumers and supporting ethical claim communication [5]. 

Ayush GCP: Although Ayurveda, Siddha, and Unani (ASU) systems have a long history of traditional use, there is a growing need for scientific validation of their safety and efficacy to ensure universal acceptance and to build confidence among practitioners, regulators, and end users. Reliance solely on long-standing traditional practice is no longer considered sufficient, as stakeholders increasingly demand documented, evidence-based proof of clinical performance. To meet this expectation, clinical research involving ASU interventions must adhere to the principles of GCP, ensuring that studies are ethically conducted, scientifically rigorous, and methodologically sound. Researchers, sponsors, and manufacturers are therefore required to be fully familiar with standardized clinical procedures that support the generation of objective, reproducible, and credible data. By following AYUSH GCP guidelines, ASU-based products can demonstrate validated safety and efficacy, strengthen their legitimacy, and support the development of responsible, scientifically grounded claims [6]. 

European Regulations

The COLIPA (Comité de Liaison des Associations Européennes de L'Industrie de la Parfumerie des Produits Cosmétiques et de Toilette; The European Cosmetic, Toiletry and Perfumery Association): The European Cosmetic, Product Test Guidelines for the Assessment of Human Skin Compatibility (1997) outline essential ethical standards to ensure safety and integrity in human clinical testing. Volunteers must be fully informed, selected according to defined inclusion and non-inclusion criteria, and provide written informed consent after understanding the study’s purpose, procedures, and any potential risks. Prior to exposing participants to the test product, all available safety data on the formulation and its ingredients must be thoroughly reviewed. All study procedures must comply with national regulations and, when applicable, receive approval from an independent ethical review committee comprising medical, scientific, and lay members who ensure participant protection. Throughout the study, investigators must take every precaution to avoid unnecessary or excessive skin reactions and ensure prompt management of any unexpected or adverse events, supported by appropriate medical care. Volunteers may receive reasonable compensation for their time and inconvenience without influencing consent. Adhering to these ethical requirements forms a critical regulatory foundation, ensuring that any data generated to support a clinically proven claim is both scientifically credible and ethically sound [7]. 

European Food Safety Authority (EFSA): Within the European Union, the EFSA plays a central role in the scientific evaluation of health claims made on foods, nutraceuticals, and functional products. In addition to the approved list of “general function” health claims, companies may seek authorization for new function claims by submitting a detailed scientific dossier to the Member State authorities, who then forward it to EFSA for assessment under Article 13.5 of the Regulation. These submissions often rely on newly developed scientific evidence, and applicants may request protection of proprietary data supporting their claim. EFSA’s NDA (Nutrition, Dietetic Products, and Allergies) Panel conducts a case-by-case evaluation, requiring applicants to present robust, comprehensive, and well-structured evidence for their specific product formulation. Once a dossier is submitted, EFSA is obligated to deliver its scientific opinion within five months, ensuring timely and transparent regulatory review. This process ensures that only well-substantiated, scientifically credible, and product-specific health claims are authorized for use in the European market [8].

Helsinki Declaration: According to the Helsinki Declaration (World Medical Association, 2000), all participants must be guaranteed access to the best proven prophylactic, diagnostic, or therapeutic interventions identified during the study once the trial concludes, ensuring continued ethical care beyond participation [9].

Australian Regulations

The Therapeutic Goods Administration (TGA) clearly states that terms like “clinically proven,” “clinically tested,” or “scientifically trialled” can only be used when strong scientific evidence supports the claim. This means the product must have well-designed, published, peer-reviewed clinical trials conducted on the same formulation and dose that is being advertised. Using such terms without proper evidence may mislead consumers by suggesting a level of effectiveness that has not been proven. Therefore, any product wishing to make a clinically proven claim must generate solid human clinical data that directly supports its benefits. Following these TGA requirements ensures that clinically related claims are accurate, transparent, and trustworthy, helping to build a reliable foundation for a clinically proven status [10].

Association of Southeast Asian Nations (ASEAN) Regulations 

The ASEAN guidelines apply to ten member countries: Brunei Darussalam, Cambodia, Indonesia, Laos, Malaysia, Myanmar, the Philippines, Singapore, Thailand, and Vietnam. The ASEAN Guidelines on Claims and Claims Substantiation for Health Supplements provide a structured framework to ensure that all product claims are truthful, evidence-based, and scientifically justified. According to these guidelines, any health-related or functional claim must be supported by at least one recognized form of evidence, as determined by the regulatory authority of each Member State. Acceptable evidence includes authoritative reference texts such as pharmacopoeias, monographs, reputable textbooks, and peer-reviewed scientific journals, scientific opinions from recognized scientific organizations, official evaluations from regulatory authorities, or documented history of traditional use, supported through classical literature, expert documentation, or published scholarly reports. By allowing multiple pathways for substantiation, the ASEAN framework ensures that health supplement claims are grounded in credible, verifiable information. This approach strengthens the validity of any performance or health benefit being communicated and supports the development of trustworthy, responsibly framed, clinically oriented claims in the supplement sector [11].

US FDA Guidance

The US FDA’s Evidence-Based Review System for Health Claims provides a structured and transparent approach for assessing the scientific validity of health-related statements made on foods and dietary supplements. According to this framework, the FDA may reevaluate existing health claims, including Significant Scientific Agreement (SSA) claims or Qualified Health Claims (QHCs), based on new scientific information. This re-evaluation may occur in response to a petition or through the FDA’s own initiative. As updated evidence becomes available, the FDA assesses whether revisions are needed to ensure claims remain accurate and protective of public health. New findings may justify changes such as modifying the wording of an existing claim, upgrading a qualified claim to an SSA claim, downgrading an SSA claim, or removing a claim altogether if safety concerns arise or the evidence no longer supports it. This dynamic review system ensures that health claims remain aligned with the most current and reliable scientific evidence, thereby strengthening the integrity, accuracy, and consumer trust associated with clinically oriented or benefit-driven claims [12].

Regulations of the Ministry of Health, Labour and Welfare (MHLW), Japan

In Japan, the MHLW enforces strict regulations to prevent exaggerated or misleading claims, particularly under Paragraph 2, Article 32 of the Food Promotion Law. This regulation recognizes that health-related advertisements-especially those circulated online-may present benefits without adequate scientific evidence, leading consumers to believe unsupported claims. Such misinformation can delay proper medical consultation and negatively impact public health. To protect consumers, the MHLW prohibits any claim that overstates effects, implies unproven therapeutic benefits, or presents information in a way that may mislead or create false expectations. By enforcing these restrictions, Japan ensures that only accurate, evidence-based, and responsibly communicated claims are permitted, reinforcing the need for credible scientific substantiation behind any clinically oriented or health-promoting statement [13].

Health Canada Regulation for Natural Health Products (NHPs)

In Canada, the regulation of NHPs falls under Health Canada’s Natural and Non-prescription Health Products Directorate (NNHPD), which provides detailed guidelines outlining the level of evidence required to support safety, efficacy, and product claims. These guidelines emphasize that NHPs making health or therapeutic claims must be backed by credible scientific evidence, which may include well-designed clinical trials, systematic reviews, pharmacological data, or high-quality traditional use information. When clinical studies are conducted, they must follow recognized standards for study design, participant safety, and data quality to ensure that results are both reliable and applicable to the marketed product. Health Canada assesses whether the evidence supports the specific formulation, dose, and intended use of the product before authorizing any claim. By providing structured guidance on clinical trials and evidence expectations, Canada ensures that claims associated with natural health products are scientifically justified, transparent, and protective of consumer health, thereby contributing to the overall credibility of clinically oriented claims [14].

Clinical Trial Agencies

In India, the CTRI mandates that all interventional human studies be prospectively registered to promote transparency, ethical conduct, and public accountability. Any product intending to use the term “clinically proven” must ensure that the supporting evidence originates from registered, ethically approved, and traceable clinical studies [15].

The US FDA requires that clinical trials supporting efficacy or safety claims, especially for drugs, biologics, and certain dietary supplements, must follow FDA-compliant protocols and be registered on ClinicalTrials.gov [16].

European Medicines Agency requires that clinical studies supporting therapeutic or performance claims follow the International Council for Harmonisation Good Clinical Practice (ICH-GCP) standards and be registered in the EU Clinical Trials Register (EudraCT) [17].

Agencies such as the United Kingdom’s Medicines and Healthcare products Regulatory Agency (MHRA), Health Canada, and TGA in Australia also require prospective registration of clinical trials, adherence to ethical standards, and claim substantiation through robust, reproducible, and product-specific clinical data.

Approaches to strengthen clinically proven evidence

Establishing credible and scientifically sound clinically proven claims demands a comprehensive, evidence-based, and methodologically rigorous approach. Integrating objective instrumental measurements with validated subjective assessments enhances the robustness and reliability of clinical data. Employing placebo-controlled, double-blind study designs minimizes bias and ensures statistical integrity, while the use of standardized endpoints facilitates meaningful cross-study comparisons. Inclusion of diverse populations across multiple geographies strengthens the generalizability and regulatory acceptance of outcomes. Transparency through clinical trial registration and peer-reviewed publication further upholds scientific integrity and trust. Additionally, advancements such as AI-assisted imaging, automated data capture, and digital adherence monitoring enhance precision, objectivity, and reproducibility. Collectively, these practices ensure the generation of high-quality clinical evidence that reinforces both scientific credibility and consumer confidence in clinically proven claims.

A relevant example demonstrating the complete evidence pathway for establishing clinically proven claims is a study in which a hair-growth-promoting formulation underwent both preclinical and clinical evaluation. The research began with in-vitro investigations, where the formulation showed a clear ability to enhance the proliferation and activity of human dermal papilla cells, indicating potential hair-growth benefits at the cellular level. This was followed by preclinical in vivo studies, where the product demonstrated increased follicular activity, improved hair growth, and greater follicle density in biological models. After confirming safety and biological plausibility, the formulation progressed to a controlled human clinical trial, which showed statistically significant improvements in key parameters such as hair density, thickness, and overall growth, without any notable adverse events. This stepwise approach, from laboratory testing to preclinical validation and finally human trials, highlights how comprehensive, multi-stage evidence is essential to substantiate reliable and ethically sound clinically proven claims in hair-care research [18]. 

For nutraceuticals, any claim presented as “clinically proven” must be supported by rigorous human data demonstrating measurable benefits. For instance, a nutraceutical product advertised as “clinically proven to improve vitamin D absorption by 30%” should be backed by a well-designed, controlled human trial measuring serum 25(OH)D levels, conducted across multiple regions and diverse populations to ensure generalizability [19].

These examples underline that clinical proof arises from structured scientific validation rather than isolated data points or observational feedback.

Conclusion

Attaining the designation “clinically proven” is a comprehensive, evidence-driven process that demands meticulous research, expert evaluation, and regulatory validation. Each phase, from initial in vitro studies to large-scale, multicentric human trials, forms a crucial component of a structured framework designed to ensure that product claims are transparent, reproducible, and scientifically substantiated. Consideration of factors such as geographical diversity, investigator proficiency, and adherence to standardized methodologies further strengthens the credibility and robustness of the evidence. Ultimately, a “clinically proven” claim reflects not only demonstrated efficacy and safety but also embodies a commitment to scientific integrity, ethical responsibility, and consumer trust, aligning innovation with regulatory and societal accountability. 
